# Insights into the strategy of micro-environmental adaptation: Transcriptomic analysis of two alvinocaridid shrimps at a hydrothermal vent

**DOI:** 10.1371/journal.pone.0227587

**Published:** 2020-01-10

**Authors:** Fang-Chao Zhu, Jin Sun, Guo-Yong Yan, Jiao-Mei Huang, Chong Chen, Li-Sheng He

**Affiliations:** 1 Institute of Deep-sea Science and Engineering, Chinese Academy of Sciences, Sanya, Hainan, China; 2 College of Earth and Planetary Sciences, University of Chinese Academy of Sciences, Beijing, China; 3 Department of Ocean Science, The Hong Kong University of Science and Technology, Hong Kong, China; 4 Japan Agency for Marine-Earth Science and Technology (JAMSTEC), Yokosuka, Kanagawa, Japan; Museum National d'Histoire Naturelle, FRANCE

## Abstract

Diffusing fluid at a deep-sea hydrothermal vent creates rapid, acute physico-chemical gradients that correlate strongly with the distribution of the vent fauna. Two alvinocaridid shrimps, *Alvinocaris longirostris* and *Shinkaicaris leurokolos* occupy distinct microhabitats around these vents and exhibit different thermal preferences. *S*. *leurokolos* inhabits the central area closer to the active chimney, while *A*. *longirostris* inhabits the peripheral area. In this study, we screened candidate genes that might be involved in niche separation and microhabitat adaptation through comparative transcriptomics. The results showed that among the top 20% of overexpressed genes, gene families related to protein synthesis and structural components were much more abundant in *S*. *leurokolos* compared to *A*. *longirostris*. Moreover, 15 out of 25 genes involved in cellular carbohydrate metabolism were related to trehalose biosynthesis, versus 1 out of 5 in *A*. *longirostris*. Trehalose, a non-reducing disaccharide, is a multifunctional molecule and has been proven to act as a protectant responsible for thermotolerance in *Saccharomyces cerevisiae*. Putative positively selected genes involved in chitin metabolism and the immune system (lectin, serine protease and antimicrobial peptide) were enriched in *S*. *leurokolos*. In particular, one collagen and two serine proteases were found to have experienced strong positive selection. In addition, sulfotransferase-related genes were both overexpressed and positively selected in *S*. *leurokolos*. Finally, genes related to structural proteins, immune proteins and protectants were overexpressed or positively selected. These characteristics could represent adaptations of *S*. *leurokolos* to its microhabitat, which need to be confirmed by more evidence, such as data from large samples and different development stages of these alvinocaridid shrimps.

## Introduction

Deep-sea hydrothermal vents are highly dynamic and unstable, both temporally and spatially. Fluids emitted from these vents, at temperatures ranging from approximately 20°C to as high as 407°C [1], mix directly with ambient seawater (~2°C) and, thus, create steep thermal and chemical gradients. The vent-associated fauna exhibits clear zonation patterns that are consistent with the physico-chemical gradients [2]. Among the factors that affect the species distribution around hydrothermal vents, the temperature and sulphides always play predominant roles [3, 4].

In the hydrothermal fields of the Okinawa Trough, *Shinkaicaris leurokolos* (Alvinocarididae, Rimicaridinae) and *Alvinocaris longirostris* (Alvinocarididae, Alvinocaridinae) usually co-exist sympatrically but occupy distinct microbiotopes according to *in situ* observations [5]. For example, at the Iheya North Knoll in the middle Okinawa Trough, the fauna directly influenced by vent activity can be divided into four zones based on thermal conditions. Among the endemic crustaceans, *S*. *leurokolos* inhabits the central zone (defined as zone 2, 0.2–0.8 m from vent) together with the squat lobster *Shinkaia crosnieri*, while *A*. *longirostris* mainly inhabits the peripheral zone (zone 4, >2.5 m from the vent), far away from the active chimney, as do *Bathymodiolus platifrons* mussels. The area within a 0.2 m radius of the vent is considered zone 1, and the transitional area (0.8–2.5 m away from vent) between zone 2 and zone 4 is defined as zone 3 [6]. *Shinkaicaris leurokolos* exhibits a similar microhabitat preference to *Rimicaris exoculata* (Alvinocarididae, Rimicaridinae) [7]. Adult *R*. *exoculata* prefers to inhabit areas with temperatures in the range of 10–25°C, and swarms of this species may tolerate occasional heat shocks that exceed its maximum critical temperature (33–38.5±2°C) [8]. For *A*. *longirostris*, the ambient temperature is approximately 3–4°C in both hydrothermal vents and cold seeps [9]. An experiment showed that a higher optimal temperature (10–20°C) is required for *S*. *leurokolos* to reach the maximum hatching rate of its embryos than for *A*. *longirostris* (10°C) under atmospheric pressure [10]. In addition, the morphological trends of these species suitable for different vent microhabitats have been revealed. *S*. *leurokolos* and *R*. *exoculata*, which occur in the vicinity of vent fluids, have evolved a degenerate rostrum and reduced external spines, both of which reduce the impact of strong turbulent fluid flows; they also have a dorsal organ that is used for detecting dim light emitted from the vents inside their carapaces; however, *A*. *longirostris* does not have dorsal organs, and its rostrum and spines are well developed [11].

Studies have been performed to investigate the mechanisms of environmental adaptation in the vent fauna in comparison with their shallow-water relatives. The expression levels of metal-binding proteins (metallothioneins) and the activities of antioxidant enzymes (such as superoxide dismutase, catalase, and glutathione peroxidase) show significant differences between vent and coastal shrimps. These genes are thought to be associated with heavy metal detoxification [12, 13]; the expression of heat shock proteins increases in *R*. *exoculata*, the crab *Chaceon affinis*, and the annelid *Paralvinella grasslei*, following an acute heat stimulus in the laboratory [14–16]. In recent years, large-scale gene profiles of vent-endemic invertebrates such as shrimp (*Rimicaris* sp.), mussel (*Bathymodiolus platifrons*) and tubeworms (*Branchipolynoe pettiboneae*, *Lepidonotopodium* sp.), have been analysed by next-generation sequencing [9, 17–19]. Consequently, a group of genes involved in sulphur metabolism, immune defence, antioxidation and detoxification have been successfully identified as being associated with environmental adaptation. However, in addition to the dramatic changes between deep-sea and shallow-sea regions, physico-chemical characteristics also vary significantly at a finer scale around vents. Zonation may induce variable physiological and biochemical adaptations, even for the same species from different microhabitats in a single hydrothermal field [20]. Thus far, the strategies for coping with fine-scale environmental fluctuations within the deep-sea vent fauna are still unknown.

In this study, we assembled the transcriptomes of *A*. *longirostris* and *S*. *leurokolos*, compared highly expressed genes and identified positively selected genes, providing preliminary clues about the genetic basis of the microhabitat adaptation of hydrothermal alvinocaridid shrimps.

## Materials and methods

### Ethical statement

This study does not involve endangered or protected species. Sample collection was conducted in the Japanese exclusive economic zone by a Japanese government research vessel. No specific permission was required for the sampled location.

### Sample collection and sequencing

Specimens of *A*. *longirostris* and *S*. *leurokolos* samples were collected from the Sakai hydrothermal vent field (27˚31.4749' N, 126˚59.021' E; depth = 1,550 m) in the middle Okinawa Trough by the JAMSTEC ROV *KAIKO Mk-Ⅳ* during R/V *KAIREI* cruise KR15-17 in November 2015 (PI: Hiroyuki Yamamoto) [21]. After being brought on board, the specimens were immediately preserved in RNA*later* stabilization solution (Invitrogen, USA) at 4°C overnight, and then transferred to -80°C for long-term storage. Two specimens of each species were used for analysis: one for transcriptome sequencing and the other for absolute quantitative real-time PCR (qPCR). Total RNA was extracted from the dissected cephalothorax and pleon using TRIzol reagent (Invitrogen, USA). The quality and quantity of the RNA were examined by agarose gel electrophoresis and with a Qubit 2.0 Fluorometer (Invitrogen, USA). Then, cDNA libraries were constructed and sequenced on the Illumina HiSeq 4000 platform at Novogene (Beijing, China).

### *De novo* transcriptome assembly

The quality of 150 bp paired-end reads was assessed by FastQC v0.10.1 (http://www.bioinformatics.babraham.ac.uk/projects/fastqc/). Contaminated adapters and poor-quality bases were trimmed using Trimmomatic-0.36 in paired-end mode [22]. Bases at both ends of the reads were cut off if the quality score was less than 5. Then, the reads that would be cut if the average quality dropped below 15 were scanned in a 4-base-wide sliding window. Finally, reads of less than 36 bases were removed. The Trinity v2.3.2 software package was utilized to assemble clean reads into putative transcripts with the minimum k-mer coverage set to 2 and the other parameters set to default [23]. The completeness of each transcriptome assembly was evaluated by using BUSCO v3.0.2 and Arthropda OrthoDB9 [24]. To remove redundant isoforms, only the longest transcript of each gene set was selected as a unigene.

### Phylogenetic analysis

Mitochondrial *cytochrome c oxidase subunit I* (*COI*) and *16S rRNA* genes were separately used for phylogenetic analysis. The full-length *COI* and *16S rRNA* genes of ten alvinocaridid species were downloaded from the NCBI database, and the pandalid shrimp *Heterocarpus ensifer* (Pandalidae) was used as an outgroup. The downloaded genes were searched against the unigenes using the Blastn program (Blast+ v2.5.0) to retrieve the assembled COI and 16S sequences. Multiple sequence alignment was performed using the MAFFT v7.294b program [25], and the aligned sequences were subsequently trimmed using the trimAl v1.4 tool [26]. Total lengths of 1,534 bp (COI) and 1,303 bp (16S) were reserved for the construction of maximum-likelihood phylogenetic trees. The TIM2+F+I+G4 model and TIM3+F+G4 model were selected for the COI and 16S rRNA sequences using ModelFinder, respectively. The phylogenetic trees were inferred by using IQ-TREE version 1.6.12 with 1,000 ultrafast bootstraps [27].

### Annotation of protein-coding genes

TransDecoder v3.0.1 was used to predict candidate open reading frames (ORFs) from unigenes with homology to known proteins via Blast or pfam searches (https://github.com/TransDecoder/TransDecoder). All predicted protein sequences were searched against the NCBI non-redundant (nr, downloaded in 08/03/2017) protein database via Blastp alignment (Blast+ v2.5.0) with an e-value cutoff of 1e-05. Conserved protein domains were identified by searching the Pfam 30.0 database using InterProScan v5.22 [28]. Gene Ontology (GO) annotation was implemented with Blast2Go Basic v5.2.5 [29]. KEGG pathway annotation was carried out with the online tool KEGG Automatic Annotation Server (http://www.genome.jp/tools/kaas/).

### Comparison of highly expressed genes

Gene expression levels measured as transcripts per million (TPM) values were calculated with RSEM 1.3.0 [30]. The top 20% of highly expressed proteins were extracted and then classified into particular groups based on the annotated GO terms by using the online tool WEGO 2.0 [31]. The percentages of each gene group were compared between *A*. *longirostris* and *S*. *leurokolos*. Pearson’s Chi-square test was applied for 2×2 matrixes if all the expected gene numbers were greater than 5. A *p*-value < 0.05 indicated a significant difference.

### Positive selection analysis

Orthogroups of pairwise species were predicted using InParanoid 4.1 with default parameters [32]. The coding sequences of *Daphnia pulex* were obtained from Ensembl Genomes and served as an outgroup [33]. Only orthogroups with single-copy genes (one to one orthologue pairs) were retained for positive selection analysis. For each single-copy orthogroup, protein-coding sequence alignment was implemented with ParaAT v2.0, in which the multiple sequence alignment program was specified as MAFFT, and both aligned codons with gaps and mismatched codons were removed [34]. The ratio of the number of nonsynonymous substitutions per nonsynonymous site (Ka) to the number of synonymous substitutions per synonymous site (Ks) was calculated using KaKs Calculator 2.0 with the model-averaging method [35]. Multiple testing correction was performed via false discovery rate (FDR) estimation. Orthologous pairs with an FDR>0.05, Ks<0.01, Ks>1, or Ka>1 were discarded [36]. Ka/Ks>1 indicated strong positive selection. Ka/Ks>0.5 was also used as a less conservative cut-off that had proven to be useful for identifying positively selected genes (PSGs) [37]. The functions of candidate PSGs with Ka/Ks>0.5 were enriched using TBtools under the threshold of an adjusted *p*-value < 0.05 [38].

### Quantitative real-time PCR

Approximately 2 μg of total RNA was used for cDNA synthesis by using High Capacity cDNA Reverse Transcription Kits (Applied Biosystems, USA), and contaminating DNA was removed by using the TURBO DNA-free Kit (Ambion, USA). The cDNA products were diluted 10-fold and used as templates. Primer pairs were designed with the NCBI on-line tool Primer-BLAST (S1 Table). The target gene fragment was amplified using PrimeSTAR HS DNA Polymerase (Takara, Japan) and cloned into the pMD18-T vector (Takara, Japan). Then, the recombinant plasmid was transformed into DH5α competent cells and positive clones were sent to BGI for Sanger sequencing. The quantification results for the transcriptomes were validated by absolute qPCR using TB Green Premix Ex Taq Ⅱ (Takara, Japan) and the StepOnePlus Real-Time PCR system (Applied Biosystems, USA). The recombinant plasmid was extracted using a TIANprep Mini Plasmid Kit (TIANGEN, China). A standard curve was generated with serial 10-fold dilutions of the recombinant plasmid. The real-time PCR mixture (20 μl) contained 10 μl of TB Green Premix Ex Taq II (2×), each of forward and reverse primers at 0.4 μM, 0.4 μl of ROX reference dye, and 2 μl of diluted cDNA. The amplification program was as followes: 95°C for 30 s, followed by 40 cycles of 95°C for 5 s and 60°C for 30 s. All samples were tested in three technical replicates. Putative homologous genes of trehalose-6-phosphate synthases (TPSs) were also confirmed by PCR. The sequenced data were searched against predicted TPSs in transcriptomes with Blastx and aligned with Clustal Omega [39].

## Results

### Transcriptome assembly and annotation

A total of 33,324,253 and 39,266,315 pairs of raw reads were generated for *A*. *longirostris* and *S*. *leurokolos*, respectively. After quality filtering, 82.98% and 82.41% the raw reads were retained for *de novo* transcriptome assembly, which generated 158,408 trinity transcripts for *A*. *longirostris* and 173,354 for *S*. *leurokolos*. Accordingly, the assembled transcripts had average lengths of 757.75 and 677.35 bp, with N50 lengths of 1,460 and 1,198 bp for *A*. *longirostris* and *S*. *leurokolos* (Table 1). When aligned with 1,066 benchmarking universal single-copy orthologues (BUSCOs) from arthropods, 90.7% complete BUSCOs were found to be present in the transcriptome of *A*. *longirostris* and 89.6% in *S*. *leurokolos* (S2 Table).

**Table 1:** Information on the *de novo* transcriptome assembly.

	*A*. *longirostris*	*S*. *leurokolos*
Data filtering:		
Number of read pairs	33,324,253	39,266,315
Number of clean read pairs	27,651,484 (82.98%)	32,360,581 (82.41%)
Trinity assembly:		
Number of transcripts	158,408	173,354
Percent GC	39.33%	39.23%
Contig N50	1,460	1,198
Median contig length (bp)	356	340
Average contig length (bp)	757.75	677.35
Number of unigenes	129,409	143,754

Excluding redundant isoforms, 129,409 and 143,754 unigenes were retained for *A*. *longirostris* and *S*. *leurokolos*, respectively. Then, 28,782 and 34,390 ORFs with a minimum length of 300 bp were predicted for *A*. *longirostris* and *S*. *leurokolos*, respectively. By database searching, 20,730 (for *A*. *longirostris*) and 23,720 (for *S*. *leurokolos*) predicted proteins were annotated in at least one database. In particular, 20,581 and 23,548 sequences returned significant hits in the nr database (Table 2). The top-hits species distribution showed that most of the predicted protein sequences were similar to proteins from the amphipod *Hyalella azteca*, indicating that no obvious contamination was present in both of the assembled transcriptomes (S1 Fig).

**Table 2:** Annotation of protein-coding sequences.

	*A*. *longirostris*	*S*. *leurokolos*
Predicted ORFs	28,782	34,390
NCBI nr	20,409 (70.9%)	23,392 (68.0%)
Gene Ontology (GO)	16,898 (58.7%)	19,559 (56.9%)
KEGG Ortholog (KO)	6,738 (23.4%)	6,835 (19.9%)
Pfam	14,863 (51.6%)	16,680 (48.5%)
Annotated in all databases	4,305 (15.0%)	4,388 (12.8%)
Annotated at least in one database	20,730 (72.0%)	23,720 (69.0%)

### Phylogenetic analysis of shrimp

**Fig 1:**
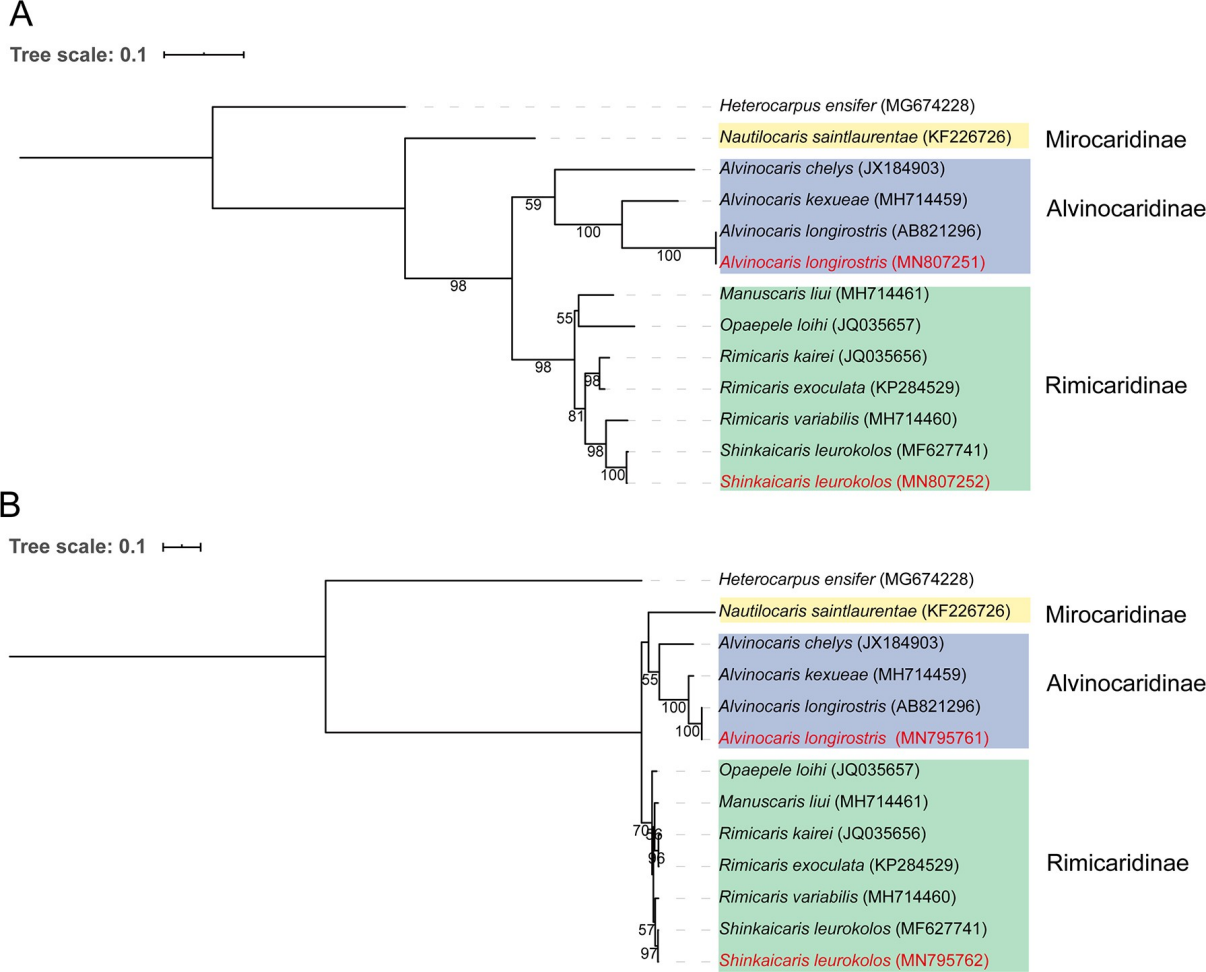
**Maximum-likelihood phylogenetic tree of alvinocaridid shrimps based on *COI* (A) and *16S rRNA* (B) genes.** The sequences in red are from this study. Statistical supports is indicated as bootstrap values, and the values of less than 50 are omitted. Subfamilies are masked by different colours: Alvinocaridinae (blue), Mirocaridinae (yellow), Rimicaridinae (green). *Heterocarpus ensifer* (family: Pandalidae) serves as the outgroup. Accession numbers are labeled in parentheses. The tree scale bar represents the number of expected substitutions per site.

Both the 16S rRNA and COI nucleotide sequences of *A*. *longirostris* used in this study shared 100% identity with an individual collected from the Hatoma Knoll in the southern Okinawa Trough (accession number of the mitochondrial genome: AB821296), while the 16S rRNA and COI nucleotide sequences of *S*. *leurokolos* shared 99.85% and 99.74% identity with a reported sample from the middle Okinawa Trough (accession number of the mitochondrial genome: MF627741). Their phylogenetic relationships with other alvinocaridid shrimps were reconstructed based on the *16S rRNA* and *COI* genes, respectively. The two trees displayed similar topologic structures. *S*. *leurokolos* clustered with the *Opaepele*, *Manuscaris* and *Rimicaris* genera, and they formed the Rimicaridinae subfamily clade. Then, this clade was separated from the *Nautilocaris* and *Alvinocaris* genera (Fig 1).

### Gene family-based comparison

After quantification, 5,761 and 6,879 genes ranked in the top 20% of highly expressed genes in *A*. *longirostris* and *S*. *leurokolos*, respectively. Among these genes, 4,144 and 4,982 successfully returned GO annotations (S3 Table). At GO level 2, the highly expressed genes showed a similar distribution for *A*. *longirostris* and *S*. *leurokolos*, most of which were concentrated in binding, metabolic process, cellular process and catalytic activity (S2 Fig).

The percentages of each gene group among the top 20% of highly expressed genes were compared between *A*. *longirostris* and *S*. *leurokolos*. Notably, there was a higher percentage of genes involved in cellular carbohydrate metabolism in *S*. *leurokolos* compared to *A*.*longirostris* (Table 3). There were 23 genes involved in this category for *S*. *leurokolos* compared to 5 genes for *A*.*longirostris*. Moreover, 15 out of 23 genes were annotated as TPSs, versus only 1 out of 5 genes in *A*. *longirostris*. The genes involved in structural components, including the extracellular matrix, integral component of membrane and cell cortex groups, were also more abundant in *S*. *leurokolos* than in *A*. *longirostris*. A higher percentage of genes involved in ribosome and translation was also observed in *S*. *leurokolos*. In addition, two groups of genes related to sulphate metabolism presented a higher proportion in *S*. *leurokolos*. One group was related to sulfuric ester hydrolases, mainly including arylsulfatase A, arylsulfatase B and N-acetylgalactosamine-6-sulfatase. The other group was related to sulfotransferases and was composed of carbohydrate sulfotransferases 9 and 11, and sulfotransferase 1C4. In contrast, two gene groups were over-represented in *A*. *longirostris*: NAD+ ADP-ribosyltransferase activity (poly (ADP-ribose) polymerase (PARP)) and cysteine-type peptidase (mainly cathepsin and ubiquitin carboxyl-terminal hydrolase) (Table 3).

**Table 3:** GO families with significant differences between *A*. *longirostris* and *S*. *leurokolos*.

GO ID	GO terms	Gene Number^a^		Gene Ratio (%)^b^		*P* value^c^
		*A*. *longirostris*	*S*. *leurokolos*	*A*. *longirostris*	*S*. *leurokolos*	
Biological Process						
GO:0006412	translation	164	270	2.847	3.925	0.001
GO:0044262	cellular carbohydrate metabolic process	5	23	0.087	0.334	0.003
GO:0032507	maintenance of protein location in cell	5	16	0.087	0.233	0.045
Cellular Component						
GO:0031012	extracellular matrix	10	26	0.174	0.378	0.032
GO:0016021	integral component of membrane	469	647	8.141	9.405	0.013
GO:0005840	ribosome	137	245	2.378	3.562	0.000
GO:0005938	cell cortex	7	22	0.122	0.320	0.020
Molecular Function						
GO:0008484	sulfuric ester hydrolase activity	6	18	0.104	0.262	0.043
GO:0016491	oxidoreductase activity	229	323	3.975	4.695	0.048
GO:0008146	sulfotransferase activity	10	27	0.174	0.392	0.023
GO:0005509	calcium ion binding	99	158	1.718	2.297	0.022
GO:0008234	cysteine-type peptidase activity	56	44	0.972	0.640	0.036
GO:0003950	NAD+ ADP-ribosyltransferase activity	16	7	0.278	0.102	0.021

a: indicates the gene number in each GO group.

b: indicates the gene percentage calculated as the gene number of each GO group divided by the total number of the top 20% of highly expressed genes.

c: indicates the value of Pearson’s Chi-square tests, where a *p*-value<0.05 indicates a significant difference.

**Fig 2:**
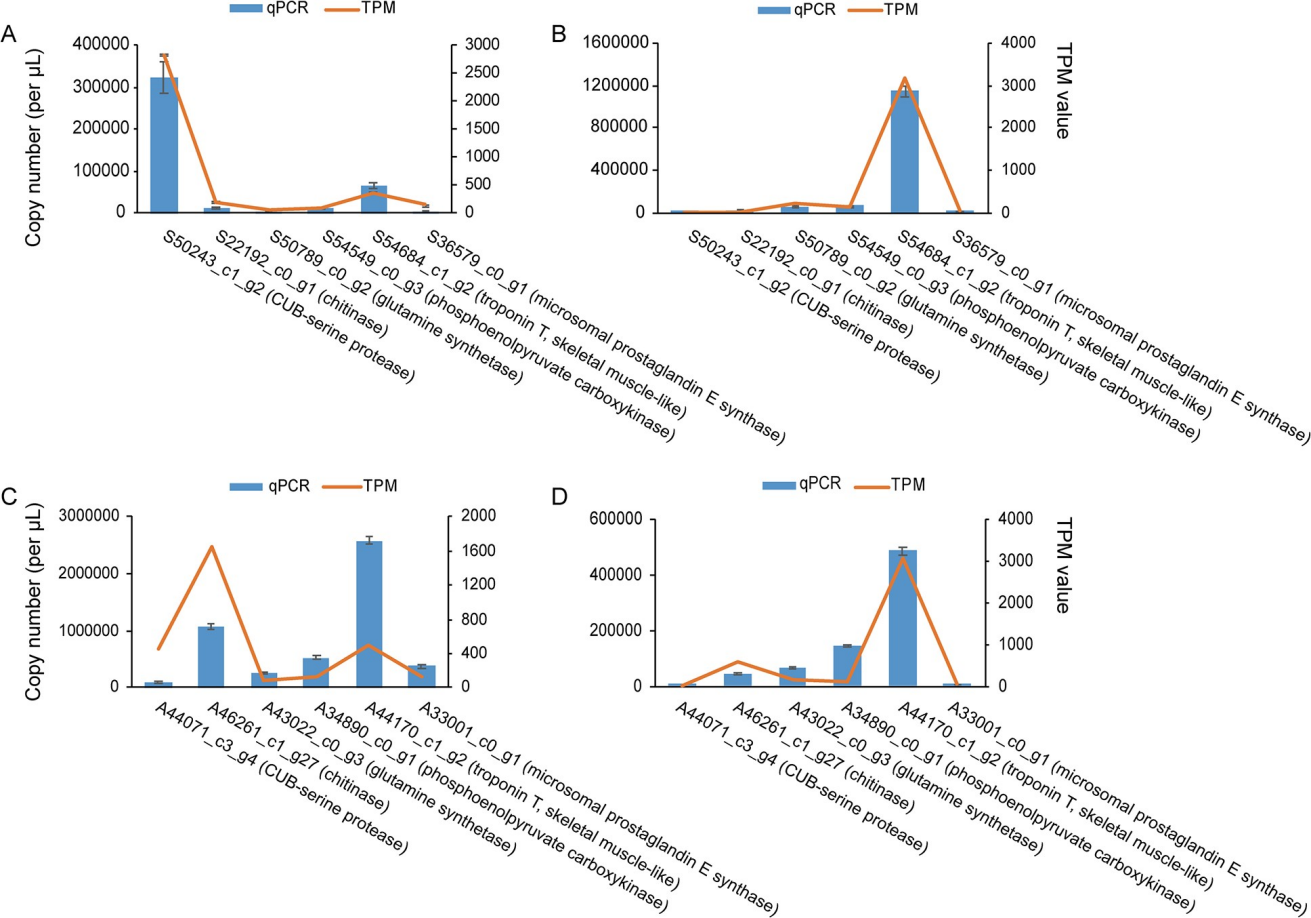
Validation of gene expression by absolute qPCR. Six single-copy orthologues for each species were randomly selected and quantified in the cephalothorax of *S*. *leurokolos* (A), abdomen of *S*. *leurokolos* (B), cephalothorax of *A*. *longirostris* (C) and abdomen of *A*. *longirostris* (D). The histograms show the gene copy number per μl (mean ± SD) with three technical replicates quantified by qPCR. The line charts show the TPM value quantified with RSEM software. Gene names are indicated on the x-axis.

To validate the RNA-seq results, six orthogroups with single-copy genes were randomly selected for absolute quantification analysis, and the variation tendencies of the gene copy numbers were consistent with the TPM values (Fig 2). Furthermore, 7 out of 15 putative TPS homologous genes from *S*. *leurokolos* were successfully amplified from the cDNA libraries, and the PCR products shared 100% amino acid identity with the corresponding TPSs assembled from the transcriptomes. The TPS segments, ranging from 100 to 325 amino acids, were aligned with TPSs from *Penaeus chinensis* and *Callinectes sapidus*. S43407_c0_g2 and S55571_c2_g2 were aligned to the glycosyltransferase family 20 domain (PF00982) of TPS from *Penaeus chinensis* (residues 7–483), while the other five unigenes were highly similar (with 59.90~81.14% sequence identity in amino acid level) to the trehalose-phosphatase domain (PF02358, residues 520–745) (S3 Fig). The presence of a TPS sequence from *A*. *longirostris* was also confirmed.

### Positively selected genes

In total, 12,544 orthogroups were identified for *A*. *longirostris* and *S*. *leurokolos*. A total of 11,002 pairs were comprised of single-copy genes and used for positive selection analysis. After filtering the data with an FDR>0.05, Ks<0.01, Ks>1 and Ka>1, 9,114 pairs of single-copy orthogroups were finally retained. Among these orthogroups, 402 pairs of orthologous genes exhibited a Ka/Ks value greater than 0.5 (S4 Fig), and 20 pairs of orthologues presented a Ka/Ks value greater than 1. However, only four PSGs were successfully annotated in the nr database: CUB-serine protease, trypsin, collagen alpha-1(IV) chain and NAD-specific glutamate dehydrogenase (S4 Table).

These genes with Ka/Ks values > 0.5 were further analysed for functional enrichment (S4 Table). As a result, five genes possessing sulfotransferase activity and eight genes participating in chitin metabolic processes were found to be significantly enriched among the moderate PSGs. Another eight enriched PSGs exhibited carbohydrate binding activities, and six of them were lectins. The next enriched group belonged to endopeptidase, including nine serine proteinases. The antimicrobial peptides (AMPs) were enriched in the groups of molecular function regulators and extracellular regions (Table 4).

**Table 4:** Enriched positively selected genes in *S*. *leurokolos*.

Gene ID	E value	Nr annotation
sulfotransferase activity (GO:0008146)		
S50007_c0_g1	1.87E-65	protein-tyrosine sulfotransferase [*Tribolium castaneum*]
S54388_c0_g1	4.00E-103	sulfotransferase 1C4-like [*Hyalella azteca*]
S52224_c0_g2	3.24E-80	galactosylceramide sulfotransferase-like [*Hyalella azteca*]
S51757_c0_g1	5.26E-50	carbohydrate sulfotransferase 11-like [*Hyalella azteca*]
S53761_c0_g1	9.83E-68	carbohydrate sulfotransferase 1-like [*Hyalella azteca*]
carbohydrate binding (GO:0030246)		
S48621_c1_g10	4.57E-30	lectin B isoform 2, partial [*Marsupenaeus japonicus*]
S49766_c0_g1	1.71E-85	C-type lectin 3 [*Fenneropenaeus merguiensis*]
S43185_c0_g1	1.10E-08	C-type lectin [*Procambarus clarkii*]
S45873_c1_g1	1.97E-31	C-type lectin 2 [*Marsupenaeus japonicus*]
S46700_c0_g1	3.15E-24	C-type lectin-like domain-containing protein [*Portunus trituberculatus*]
S54525_c4_g1	3.55E-37	lectin B isoform 2, partial [*Marsupenaeus japonicus*]
S45454_c0_g1	8.76E-17	natterin-4-like [*Acropora digitifera*]
S53662_c1_g1	3.79E-101	scavenger receptor C [*Marsupenaeus japonicus*]
chitin metabolic process (GO:0006030), extracellular region (GO:0005576)		
S51908_c0_g1	0.00E+00	chitinase 10 [*Locusta migratoria*]
S52856_c0_g4	1.34E-15	peritrophin-44-like protein [*Eriocheir sinensis*]
S53991_c0_g1	0.00E+00	mucin-3A [*Camponotus floridanus*]
S54047_c0_g1	3.11E-07	mucin-5AC, partial [*Orussus abietinus*]
S24173_c0_g1	NA	NA
S51404_c1_g2	NA	NA
S51697_c0_g1	NA	NA
S48582_c0_g1	NA	NA
molecular function regulator (GO:0098772)		
S46156_c0_g2	2.35E-36	antimicrobial peptide type 2 precursor IIc [*Pandalopsis japonica*]
S38510_c0_g1	4.08E-25	antimicrobial peptide type 1 precursor Ie [*Pandalopsis japonica*]
S54779_c0_g10	2.81E-29	antimicrobial peptide type 2 precursor IIc [*Pandalopsis japonica*]
S47957_c0_g1	2.26E-127	serine/threonine-protein phosphatase 2A activator-like [*Hyalella azteca*]
S43281_c0_g1	1.46E-14	serine/threonine-protein kinase samkC [*Hyalella azteca*]
S41765_c0_g3	3.18E-107	alpha-2-macroglobulin [*Macrobrachium rosenbergii*]
S1809_c0_g1	2.62E-10	macroglobulin [*Palaemon carinicauda*]
S53590_c2_g2	9.01E-17	annexin A11 isoform X1 [*Salmo salar*]
S51629_c0_g1	4.13E-178	ADP-ribosylation factor GTPase-activating protein 2-like isoform X1 [*Hyalella azteca*]
S53169_c0_g1	0.00E+00	PH and SEC7 domain-containing protein 1 isoform X1 [*Vollenhovia emeryi*]
S21248_c0_g1	1.20E-20	puratrophin-1, partial [*Chlamydotis macqueenii*]
S49552_c0_g1	NA	NA
endopeptidase activity (GO:0004175)		
S48676_c0_g1	5.55E-65	trypsin-like serine proteinase [*Scylla paramamosain*]
S53405_c0_g1	3.18E-15	caspase [*Eriocheir sinensis*]
S49898_c0_g1	0.00E+00	lon protease homolog 2, peroxisomal-like [*Crassostrea gigas*]
S48935_c0_g1	8.25E-07	CUB-serine protease [*Panulirus argus*]
S42913_c0_g1	8.78E-35	trypsin 3A1-like [*Aedes albopictus*]
S51196_c0_g1	7.32E-140	serine protease [*Macrobrachium rosenbergii*]
S45313_c0_g1	3.83E-37	chymotrypsin-like protein [*Daphnia pulex*]
S53118_c0_g2	9.09E-59	trypsin [*Euphausia superba*]
S43774_c0_g1	4.44E-49	tryptase-like [*Ictalurus punctatus*]
S50445_c0_g1	2.18E-62	trypsin-1-like [*Dendroctonus ponderosae*]
S54451_c0_g2	3.42E-51	SpAN-like protein, partial [*Rimicaris exoculata*]
S55389_c2_g5	2.90E-59	protein SpAN-like isoform X2 [*Hyalella azteca*]
S54596_c0_g2	9.78E-95	protein SpAN-like [*Hyalella azteca*]
S46490_c0_g1	1.24E-07	ADAM metalloprotease, partial [*Marsupenaeus japonicus*]
extracellular region (GO:0005576)		
S53590_c2_g2	9.01E-17	annexin A11 isoform X1 [*Salmo salar*]
S27614_c0_g1	1.69E-45	serine proteinase inhibitor 8 [*Penaeus monodon*]
S1809_c0_g1	2.62E-10	macroglobulin [*Palaemon carinicauda*]
S43856_c0_g1	3.18E-12	putative insulin-like protein growth factor binding protein [*Tityus obscurus*]
S51978_c0_g6	1.32E-34	serum amyloid A-5 protein-like isoform X1 [*Branchiostoma belcheri*]
S45464_c0_g1	1.04E-95	putative endothelial lipase [*Hyalella azteca*]
S49552_c0_g1	1.13E-27	oxidoreductase NAD-binding domain-containing protein 1 isoform X4 [*Cavia porcellus*]
S50357_c0_g1	7.96E-69	fibrinogen C domain-containing protein 1-A-like isoform X1 [*Hyalella azteca*]
S38510_c0_g1	4.08E-25	antimicrobial peptide type 1 precursor Ie [*Pandalopsis japonica*]
S46156_c0_g2	2.35E-36	antimicrobial peptide type 2 precursor IIc [*Pandalopsis japonica*]
S54779_c0_g10	2.81E-29	antimicrobial peptide type 2 precursor IIc [*Pandalopsis japonica*]
S3003_c0_g1	8.40E-13	oxygenase [*Oplophorus gracilirostris*]
S51450_c0_g1	8.12E-64	oxygenase [*Oplophorus gracilirostris*]
S46892_c0_g2	1.76E-45	oxygenase [*Oplophorus gracilirostris*]

## Discussion

How organisms adapt to deep-sea environments has always been an interesting topic. The subject of adaptation to the microenvironment in special areas such as hydrothermal vent fields is easy to be ignore but important. In this paper, two vent-endemic alvinocaridid shrimps were used as an example to illustrate the possible genes and pathways involved in microenvironmental adaptation. The protein synthesis rate has a significant impact on thermal acclimation, although the relationship between them is complex [40, 41]. Proteins are usually vulnerable to elevated temperatures because they maintain their function within only a narrow range of temperatures. It has been demonstrated that *in vitro* high temperatures inhibit mRNA translation by suppressing Met-tRNA synthetase activity [42]. Decreased protein turnover reduces metabolic sensitivity to environmental change [43]. Therefore, active protein synthesis may be a compensation mechanism to balance the protein turnover. In this study, genes associated with ribosomes and translation were highly expressed, indicating more active protein synthesis in *S*. *leurokolos* compared to *A*. *longirostris*. In the group of cellular carbohydrate metabolism of *S*. *leurokolos*, more than 60% of genes were annotated as TPSs, which were key enzymes for trehalose biosynthesis. Trehalose, a non-reducing disaccharide, is a multifunctional molecule that plays important roles in sugar metabolism, stress recovery, chitin synthesis and other biological processes [44]. Trehalose is also a protectant responsible for thermotolerance, as demonstrated in *Saccharomyces cerevisiae* [45]. Functionally, it acts as a chemical co-chaperone to delay protein degradation and aggregation, possibly due to the preferential formation of the peptide-trehalose hydrogen bond [46, 47]. The presence of TPS homologs was validated by PCR, and we inferred that there were at least four different *TPS* genes in *S*. *leurokolos*, according to sequence alignment. The TPSs existing in deep-sea invertebrates have been poorly investigated, and the functions of different TPSs within the same species are still unclear. However, as a primary enzyme in trehalose synthesis, an increase in trehalose might help *S*. *leurokolos* to cope with temperature variation and other stresses.

Basic structural proteins such as extracellular matrix, integral component of membrane and cell cortex proteins displayed distinct expression patterns between *S*. *leurokolos* and *A*. *longirostris*. Among these proteins, the basement-membrane collagen alpha-1(IV) chain protein was found to be under particularly strong positive selection. The deep-sea polychaetous annelids *Alvinella pompejana* and *Riftia pachyptila* present a similar living pattern to the shrimps investigated in this study: the former inhabits the surface of chimney walls and tolerates temperatures up to 60–65°C; the latter inhabits regions with a relatively lower temperature (approximately 37°C). The thermal tolerance of *A*. *pompejana* is mainly due to interstitial collagen because of its increased proline content and hydroxylation [48]. However, in another kind of fibrillar collagen from *R*. *pachyptila*, glycosylated threonine but not 4-hydroxyproline contributes to triple helix stability [49]. All of the potential collagens in the transcriptomes of *A*. *longirostris* and *S*. *leurokolos* were identified by conserved domain searches, and the compositions of their amino acids were calculated. The results showed that the percentages of proline (12.34% in *A*. *longirostri* versus 13.80% in *S*. *leurokolos*) and threonine (5.38% versus 4.46%) in collagens were significantly different between the species (S5 Table). We infer that the thermostability of collagen from *S*. *leurokolos* differs from that of *A*. *longirostris*. Another structure-related group that was enriched in the putative PSGs of *S*. *leurokolos* was the chitin metabolic process category. Peritrophin-44 and mucin are components of the peritrophic membrane, a non-cellular structure secreted from the midgut epithelium of invertebrates. Chitinase is generally found in tissues (such as the peritrophic membrane) that either require the remodelling of chitinous structures or degradation of digested chitin [50]. In decapod crustaceans, the peritrophic membrane commonly provides an intestinal barrier that protects against mechanical and chemical damage and prevents pathogen infection [51].

Genes participating in innate immunity of invertebrates were enriched among the positively selected genes, including lectin, caspase, serine proteinase and AMP. As important pattern recognition receptors (PRRs), the crustacean lectins recognize glycans on the cell surface of invading pathogens and activate a range of immune responses [52]. However, PRRs are also required to promote the normal colonization of gut microbiota [53]. A C-type lectin from *R*. *exoculata* recognizes and agglutinates *Escherichia coli in vitro* without the inhibition of bacterial growth [54]. Commonly, AMPs directly kill pathogens by disrupting their cell membranes. However, an AMP known as coleoptericin-A from weevil selectively targets endosymbionts within bacteriocytes and controls their growth through the inhibition of cell division [55]. More importantly, it has been reported that caspases regulate endosymbiont density in deep-sea *Bathymodiolus* mussels through the mechanism of gill cell apoptosis [56]. The serine proteinases not only regulate antimicrobial peptide synthesis and prophenoloxidase activation but also mediate apoptosis-like cell death [57, 58]. Thus, the lectins, caspases, serine proteinases and AMPs as well as other innate immune molecules are also possibly involved in the management of symbiont populations. The dominant chemosynthetic bacteria associated with *A*. *longirostris* and *S*. *leurokolos* are assumed to be different because of the divergence of their carbon fixation pathways [59]. In addition, the size of the host symbiotic bacterial population varies according to the supply of free H_2_S/HS^-^ in the environment [60]. By analogy with *R*. *exoculata*, *S*. *leurokolos* probably has more abundant symbionts in its gill chambers. Therefore, the identified immune molecules may contribute to the differences between *A*. *longirostris* and *S*. *leurokolos* in terms of distinguishing different symbiotic bacteria and regulating their densities to address environmental fluctuations.

Extremely high genetic diversity of *S*. *leurokolos* was revealed in the Okinawa Trough, but *A*. *longirostris* showed low genetic diversity [61]. The *16S rRNA* nucleotide sequences obtained in our study were nearly identical (>99%) with those of previously reported *A*. *longirostris* and *S*. *leurokolos* samples, as were those of the *COI* genes [62]. However, even within the same species, variation in environmental acclimation exists between populations and phylogenetic lineages [63]. Therefore, the genes screened in our study still need to be further confirmed based on a dataset including replicate specimens for each species.

In conclusion, genes related to protein synthesis, structural components and trehalose biosynthesis might be involved in thermal acclimation, and a group of immune proteins might be involved in symbiosis preservation. The differences observed between the two species for these genes provide clues about the discrepancy in microhabitats between *A*. *longirostris* and *S*. *leurokolos*.

## Supporting information

S1 Fig Top-hit species classification of predicted proteins with nr annotation. A indicates *A*. *longirostris* and B indicates *S*. *leurokolos*.
(TIF)

S2 Fig Gene ontology distribution. The top 20% of highly expressed genes were analysed. The X-axis shows the GO terms in level 2; the y-axis shows the percentages of genes (number of a particular gene divided by total gene number) on the left and the number of genes on the right.
(TIF)

S3 Fig Multiple alignment of validated TPSs. NCBI accession number ACD74843.1 indicates TPS from *Penaeus chinensis*, and ACI12944.1 indicates TPS from *Callinectes sapidus*.
(TIF)

S4 Fig Distribution of Ka and Ks values. Dots between the y-axis and the grey line represent orthologous pairs with a Ka/Ks ratio>1, dots between the x-axis and the red line represent orthologous pairs with a Ka/Ks ratio<0.5, and dots between the red and grey lines represent a 1>Ka/Ks ratio>0.5.
(TIF)

S1 Table Primer sets used in this work. (XLSX)

S2 Table Completeness estimation of transcriptome assemblies. (XLSX)

S3 Table List of the top 20% of highly expressed genes. (XLSX)

S4 Table Orthologous genes displaying evidence of positive selection. (XLSX)

S5 Table The percentages of amino acids in collagens from *A*. *longirostris* and *S*. *leurokolos*. (XLSX)
