# Supplementary figures and images for: Insights into the strategy of micro-environmental adaptation: Transcriptomic analysis of two alvinocaridid shrimps at a hydrothermal vent
Source: PLoS One. 2020 Jan 10;15(1):e0227587. doi: 10.1371/journal.pone.0227587 (PMC6953826; doi:10.1371/journal.pone.0227587)

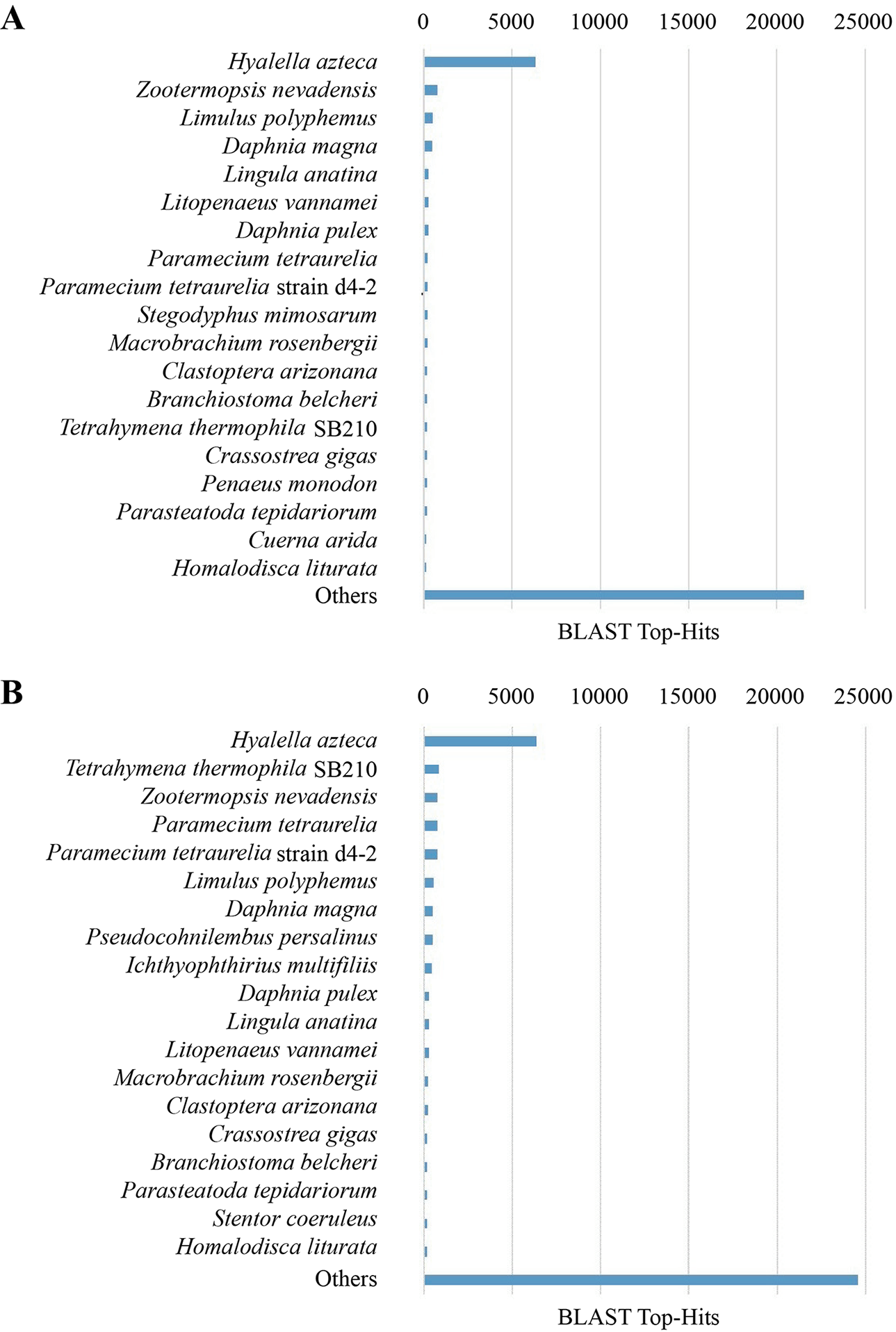

Supplement: S1 Fig — A indicates A. longirostris and B indicates S. leurokolos. (TIF) [file pone.0227587.s001.tif]

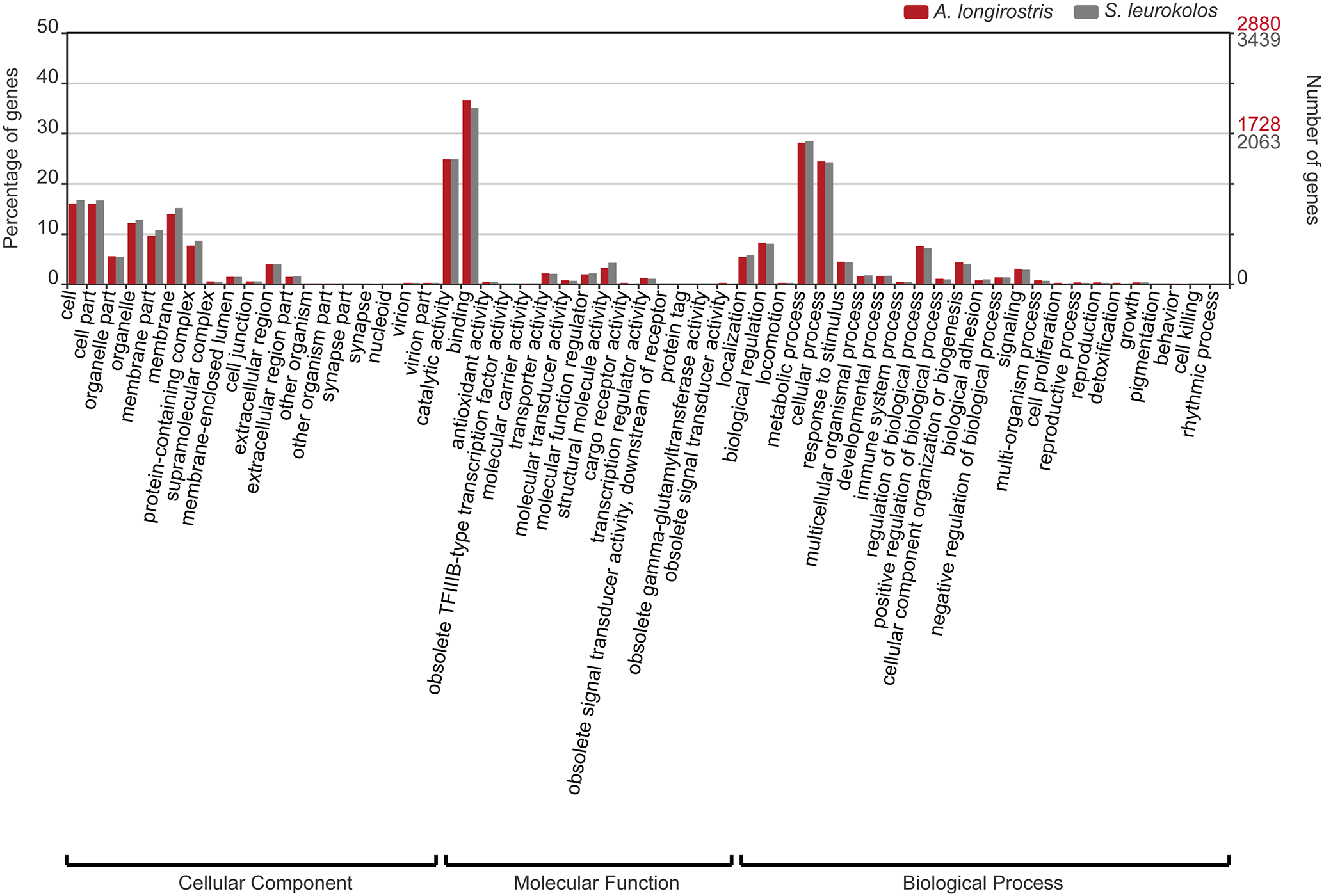

Supplement: S2 Fig — The top 20% of highly expressed genes were analysed. The X-axis shows the GO terms in level 2; the y-axis shows the percentages of genes (number of a particular gene divided by total gene number) on the left and the number of genes on the right. (TIF) [file pone.0227587.s002.tif]

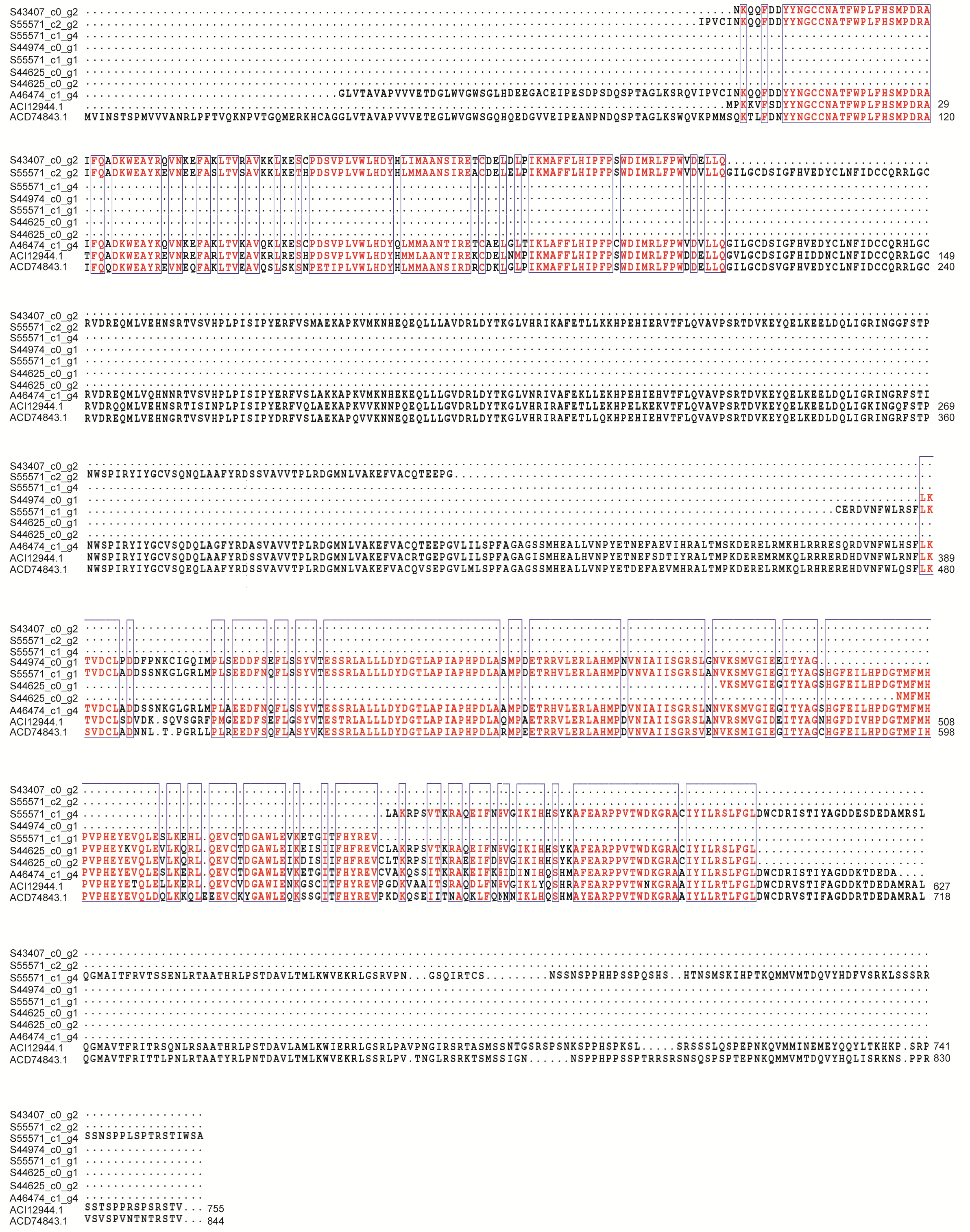

Supplement: S3 Fig — NCBI accession number ACD74843.1 indicates TPS from Penaeus chinensis, and ACI12944.1 indicates TPS from Callinectes sapidus. (TIF) [file pone.0227587.s003.tif]

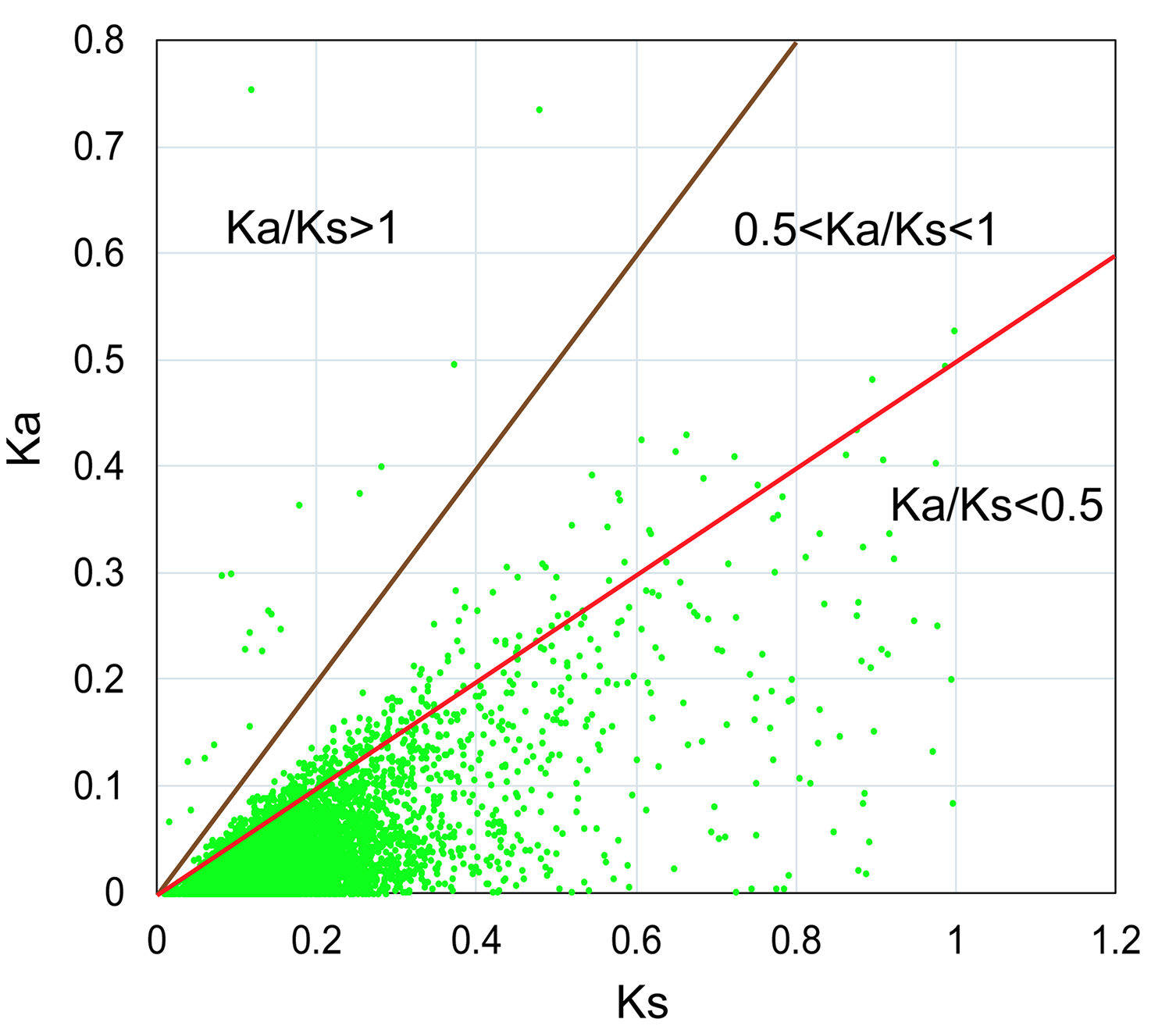

Supplement: S4 Fig — Dots between the y-axis and the grey line represent orthologous pairs with a Ka/Ks ratio>1, dots between the x-axis and the red line represent orthologous pairs with a Ka/Ks ratio<0.5, and dots between the red and grey lines represent a 1>Ka/Ks ratio>0.5. (TIF) [file pone.0227587.s004.tif]
